# New insights into the role of ID proteins in breast cancer metastasis: a MET affair

**DOI:** 10.1186/bcr3654

**Published:** 2014-05-15

**Authors:** Wee Siang Teo, Radhika Nair, Alexander Swarbrick

**Affiliations:** 1 Cancer Research Division, The Kinghorn Cancer Centre and Cancer Research Program Garvan Institute of Medical Research, 370 Victoria St, Darlinghurst, NSW 2010, Australia; 2 St Vincent's Clinical School, Faculty of Medicine, University of New South Wales, Sydney, NSW 2052, Australia

## Abstract

The establishment of lethal metastases depends on the capacity of a small number of cancer cells to regenerate a tumor after entering a target organ. Stankic and colleagues have identified a role for the inhibitor of differentiation protein, ID1, as a critical regulator of breast cancer stem-like properties and metastatic colonization. Under the control of tumor growth factor-beta signaling, ID1 induces mesenchymal-epithelial transition at the metastatic site by antagonizing the activity of the basic helix-loop-helix transcription factor Twist1. This study sheds light on mechanisms that initiate metastatic outgrowth, and strengthens the concept that epithelial-mesenchymal plasticity is crucial at different stages of metastasis.

## Background

A major focus of metastatic studies is in understanding the mechanisms by which tumor cells escape the local environment and colonize distant organs. Emerging evidence suggests that activation of the epithelial-to-mesenchymal transition (EMT) program drives metastasis [1] and is associated with a gain of mesenchymal properties and stem cell-like behavior [2,3]. It has been proposed that the reversal of EMT, the mesenchymal-to-epithelial transition (MET), is necessary for efficient metastatic colonization by allowing re-differentiation of disseminated tumor cells into an epithelial phenotype required for proliferation [1]. Although clinical reports support the concept of transient EMT-MET switches in metastasis [4], there is only limited experimental evidence for this phenomenon and few insights into the mechanisms that induce MET in metastatic sites.

## Article

A recent study from Robert Benezra’s group [5] provides evidence to support the role of a MET program for efficient metastatic growth and a mechanism controlling this process. The authors identified the inhibitor of differentiation protein, ID1, as a critical regulator of breast tumor-initiating phenotype and metastatic colonization. ID1 is a helix-loop-helix protein which acts as a dominant negative regulator of many transcription factors [6]. ID1 is overexpressed in aggressive triple-negative breast tumors [7] and is required for the metastasis of breast cancer in experimental models [8-10]. Using the immortalized human mammary epithelial cells, Stankic and colleagues [5] showed that overexpression of ID1 can generate breast cancer cells with cancer stem cell (CSC)-like properties and that these cells were characterized by a high self-renewal capacity, enrichment in CD44^high^/CD24^low^ expression, and an increased tumor-initiating potential by limiting dilution transplantation assay *in vivo*. However, during the process of metastatic colonization, ID1 induces MET in cells that had previously undergone EMT. Conditional overexpression of ID1 revealed that ID1 promotes lung metastasis by inducing MET at the metastatic site through antagonism of the basic helix-loop-helix transcription factor Twist1. Interestingly, ID1 did not act in the same manner in the primary site, where this state is controlled by the zinc finger protein Snail. Knockdown of ID1 and ID3 expression in metastasizing cells results in a failure to initiate MET and dramatically reduces lung colonization, providing strong evidence that ID proteins are required for the maintenance and propagation of disseminated tumor cells. The authors further demonstrated that ID1 expression is regulated by tumor growth factor-beta (TGF-β) and that upregulation of ID1 by TGF-β occurs only in disseminated cancer cells that had initially seeded in a mesenchymal state, suggesting that EMT is a prerequisite for subsequent ID1-induced MET during lung colonization.

## Viewpoint

This study supports the role of phenotypic plasticity in breast cancer metastasis and, in particular, the necessity of the acquisition of a MET program at the distant organs for colonization. The complex context-dependent function of ID1 allows breast cancer cells to retain their EMT-CSC-like mesenchymal phenotype during tumor initiation and metastatic dissemination, and to re-acquire their epithelial character necessary for lung colonization. Since TGF-β can derive from both tumor and host tissue stroma [11], this implies a regulation of the TGF-β-ID1-induced MET plasticity by environmental conditions and contextual signals and a possible role of ID1 in determining organ tropism.

Interestingly, the authors showed that the CSC-like properties induced by ID1 are independent of induction of EMT; this finding is contrary to the notion that CSC properties are inextricably linked to a mesenchymal state. This also corroborates recent work by our group [12] and others [13] that the canonical EMT program is not often associated with CSC-like properties, and prompts further investigations to better define the biology and differentiation state of CSCs. Although the authors have used transplanted cancer cell lines to illustrate CSC-like properties and the transient nature of the mesenchymal phenotype controlled by ID1, evidence for the existence of such CSCs and plasticity in spontaneously arising tumors *in vivo* or in human tumors remains to be determined. Furthermore, ectopic gene expression of EMT inducers and ID1 may not provide conclusive evidence of these concepts in a complex *in vivo* tumor setting. More evidence can be achieved by lineage tracing of an EMT-MET phenotype in spontaneous mouse models of metastatic cancer or inducible loss of ID1 function.

## Abbreviations

CSC: Cancer stem cell; EMT: Epithelial-to-mesenchymal transition; ID1: Inhibitor of differentiation protein; MET: Mesenchymal-to-epithelial transition; TGF-β: Tumor growth factor-beta.

## Competing interests

The authors declare that they have no competing interests.
